# Patterns of Practice Survey: Radiotherapy for Soft Tissue Sarcoma of the Extremities

**DOI:** 10.7759/cureus.6153

**Published:** 2019-11-14

**Authors:** Chan-Kyung J Cho, Charles Catton, Caroline L Holloway, Karen Goddard

**Affiliations:** 1 Surgery, University of British Columbia, Vancouver, CAN; 2 Radiation Oncology, Princess Margaret Hospital / University of Toronto, Toronto, CAN; 3 Radiation Oncology, British Columbia Cancer Agency, Victoria Centre, University of British Columbia, Victoria, CAN; 4 Radiation Oncology, British Columbia Cancer Agency, Vancouver Centre, University of British Columbia, Vancouver, CAN

**Keywords:** soft tissue sarcoma of the extremities, patterns of practice, radiotherapy for sarcoma, sarcoma

## Abstract

Background

Neoadjuvant or adjuvant radiotherapy (RT) for extremity soft tissue sarcoma (STS) confers significant local control benefit. To determine patterns of practice, a survey of RT planning practices was undertaken.

Method

Members of the Connective Tissue Oncology Society and Canadian Association of Radiation Oncology participated in this survey pertaining to general practice patterns of RT for extremity STS, patterns of contouring and planning, and use of quality control measures such as guidelines, tumor boards, and quality assurance rounds.

Results

A total of 58 radiation oncologists treating extremity STS from 12 countries responded. 89.7% work in academically affiliated centres, and 55.2% saw at least 20 cases of extremity STS per year. Most (96.7%) had access to multidisciplinary sarcoma boards (85.5% of those discussed every referred sarcoma case). 78.6% held quality assurance rounds. Most (92.9%) used planning guidelines. Pre-operative RT was used nearly twice as much as post-operative RT. CT simulation with MR fusion was used by 94.6%. Patterns of clinical target volume (CTV) contouring for both superficial and deep STS were variable. 69.8% contoured a normal soft tissue strip for extremity sarcoma, 13.5% without routine constraints and the remainder with various constraints. Most (91.1%) used 50 Gy in 25 fractions pre-operatively and 39.6% reported using post-operative RT boost for positive margins. Post-operative dose was more variable from 59.4 Gy to 70 Gy.

Conclusion

Major aspects of RT planning for extremity STS were similar among the responders, and most were academically affiliated. Over twice as many employed pre-operative as opposed to post-operative RT. There was considerable heterogeneity in use of: margins for contouring, normal soft tissue strip as an avoidance structure, and boost for positive margins. This survey shows variable patterns of practice and identifies areas that may require further research.

## Introduction

Soft tissue sarcomas (STS) are neoplasms arising from mesenchymal cells in the adipose, muscle and connective tissues. In 2014, approximately 12,000 people were diagnosed with STS in the US, with an estimated 4,740 deaths [1]. Most STS of extremity progress with localized growth in volume with invasion of surrounding tissues. Local control while preserving functionality is an important and achievable therapeutic goal for most patients. Limb-sparing surgical resection is the primary treatment for local control and may be adequate on its own for small superficial and/or low-grade tumors with adequate negative margins.

The role of pre- or post-adjuvant radiotherapy (RT) to reduce the risk of local recurrence has been well established as the standard of care for most extremity STS. The rate of local control for combined surgery and radiotherapy is significantly improved compared to that with surgery alone [2, 3]. Although both pre-operative and post-operative RT confer similar local control, progression-free and overall survival benefits, pre-operative RT has been associated with a lower total dose, smaller area of treatment, and lower risk of late toxicities such as edema, subcutaneous fibrosis, and joint stiffness [4, 5]. Currently, most extremity STS are treated by either 3D conformal or intensity-modulated radiotherapy (IMRT), and RT planning employs dose-volume histogram constraints for optimal target coverage while protecting normal structures. Although guidelines from different organizations, such as Radiation Therapy Oncology Group (RTOG), are available, detailed evidence-based recommendations for RT contouring and planning are lacking in literature [6]. For example, most protocols recommend the use of an avoidance structure that encompasses a longitudinal strip of healthy skin and subcutaneous tissue of an extremity (“normal tissue strip”), but the volumetric and dosimetric details of such structure are highly variable.

Our objective was to organize a survey of radiation oncologists who are involved in the treatment of STS of the extremities to survey patterns of practice including practice settings, quality assurance processes, contouring and planning of RT.

## Materials and methods

The survey was created using an online survey provider (SurveyMonkey.com) and was approved by the BC Cancer Research Ethics Board. It was sent to all members of the Connective Tissue Oncology Society and the Canadian Association of Radiation Oncology by e-mail with a link to the online survey and was conducted between October and November 2017. Both the invitation email and the cover page of the survey stated that only radiation oncologists and radiation oncology fellows who treat STS of the extremities were invited to participate in this survey.

This survey included a total of 26 questions with one management scenario as a question. There were multiple “skip logic” questions where the previous answer determined whether or not the subsequent, related question would be presented to the respondent. There were four reminders throughout the survey that this survey was strictly regarding high grade (French grade 2 or 3) STS of the extremities. The survey asked about the types of practice setting and experience, annual number of STS cases, availability of multidisciplinary tumor board and quality assurance process, use of guidelines, use of normal soft tissue strip as an avoidance structure, use of pre- vs. post-operative radiotherapy, RT treatment technique, and specificities of contouring. Participants were allowed to skip questions. Our sample survey is included in the appendix.

As the number of respondents varies depending on the question, the survey results are presented as a percentage of evaluable responses. Differences among the responses between superficial and deep STS clinical target volume (CTV) contouring were analyzed by Wilcoxon Signed Ranks test via SPSS 14.0 (SPSS Inc, Chicago, IL).

## Results

Practice setting

A total of 58 radiation oncologists from 12 countries who treat STS of the extremities participated in the survey. Fifty-three were radiation oncologists, and four were radiation oncology fellows. Eighteen were from the USA, 17 from Canada, six from the Netherlands, five from the UK, three from Italy, two each from Australia and Denmark, and one each from Switzerland, Saudi Arabia, Peru, Poland, and New Zealand. Years of practice showed an inverted bell curve distribution, with 19 having greater than 20 years, and another 19 having fewer than five years of practice experience. Thirty-two (55.2%) respondents indicated that they see more than 20 cases per year, while 17 (29.3%) see 10-20 cases, and nine (15.5%) see fewer than 10 cases a year. The majority (50, 86.2%) worked in an academic setting, while 6.9% worked in private and another 6.9% in a mixed setting. Thirty-two (55.2%) worked in a center with 15 or more people.

Quality assurance

Fifty-six out of 58 respondents had access to a multidisciplinary sarcoma tumor board where the management of sarcoma cases was discussed. 85.5% indicated that they discuss every case, and the rest (14.6%) discussed only complicated or challenging cases. The guidelines employed are in Table 1. When asked what type of guideline is used to plan STS extremity, 26 (46.4%) respondents reported the use of RTOG guideline, and 16 (28.6%) used local institutional guidelines. Other guidelines included: the Dutch STS guideline, National Comprehensive Cancer Network (NCCN) guideline, European Society for Medical Oncology (ESMO) guideline, Scandinavian Sarcoma Group (SSG) guideline, Children’s Oncology Group (COG) guideline, UK trial of IMRT to treat bone and soft tissue sarcoma (IMRiS) protocol, and a review paper by Haas et al. [6, 7]. Four (7.1%) respondents did not use a specific guideline. Forty-four (78.6%) respondents had quality assurance rounds to peer review treatment volumes and plans prior to therapy.

**Table 1:** Types of guidelines used for treatment of STS of the extremities. *Includes guidelines from the European Society for Medical Oncology, Children’s Oncology Group, National Comprehensive Cancer Network, Le Centre Hospitalier Universitaire Vaudois, Scandinavian Sarcoma Group, and the UK IMRiS phase II clinical trial protocol. RTOG: Radiation Therapy Oncology Group; STS: Soft Tissue Sarcoma.

Guideline	Responses (%)
RTOG guideline	26 (46.4)
Local institutional guideline	16 (28.6)
No specific guideline	4 (7.1)
Protocol by Haas et al.	2 (3.6)
Dutch STS guideline	2 (3.6)
Others*	6 (10.7)

Simulation and RT technique

The most frequently used RT technique was volumetric-modulated arc therapy (VMAT) (41.8%), followed by standard IMRT (30.9%) and 3D conformal radiotherapy (20.0%). Four respondents reported using the available techniques equally depending on the achievable plan. When contouring gross tumor volume (GTV), CT simulation with MR fusion was the most commonly used method (52 out of 55 respondents) and four respondents also used MR simulation. Among those who use MR fusion, the average degree of satisfaction regarding the fusion was 67%.

Use of pre- vs. post-operative RT

**Figure 1:**
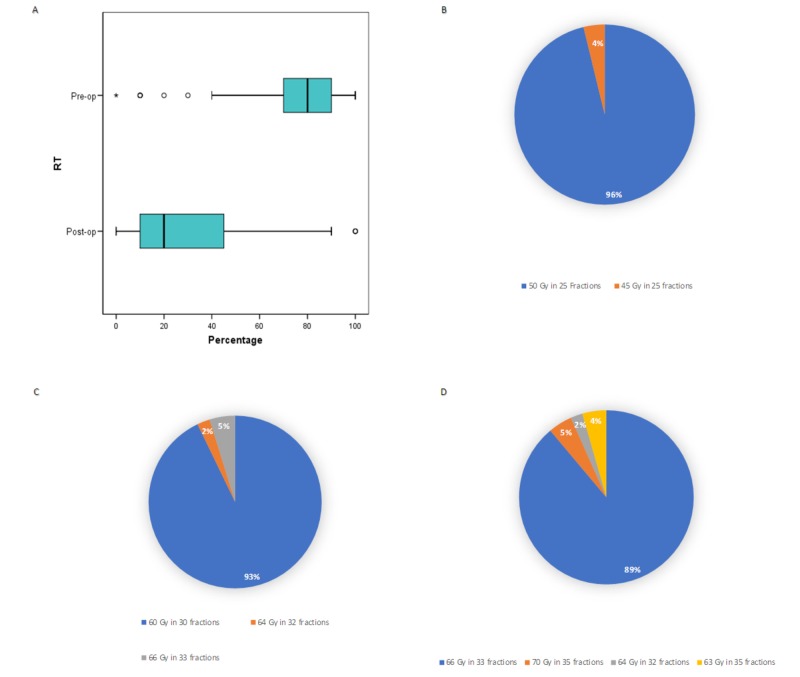
Patterns of use of pre- and post-operative radiotherapy. (A) Perceived percentage use of pre- versus post-operative radiotherapy. Dose-fractionation patterns used for treatment of extremity STS for (B) pre-operative RT, (C) post-operative RT with negative margins, and (D) post-operative RT with positive margins. For pre-operative RT, some respondents reported using 50 Gy in 25 fractions but occasionally using other dose-fractionations, such as 59.4 Gy in 33 fractions, 30 Gy in five fractions, and 25 Gy in five fractions occasionally. For post-operative RT, some reported of considering 60 Gy in 30 fractions if the target volume is large, or if the PTV is adjacent to a critical normal organ. STS: Soft tissue sarcoma; RT: Radiotherapy; PTV: Planning target volume.

Figure 1 summarizes the prevalence of pre- versus post-operative RT usage and respective dose fractionation regimens. Of 55 respondents who answered the subsequent questions, the average frequency for use of pre-operative RT was 74%. One person reported never using pre-operative RT, and six used it less than 50% of the time. The average frequency for use of post-operative RT was 31%, with four non-users and 12 (21.8%) respondents using it greater than 50% of the time. For pre-operative RT, 96.2% used 50 Gy in 25 fractions, and 3.8% used 45 Gy in 25 fractions. Two respondents reported using hypofractionation as well, such as 25 Gy in five fractions and 30 Gy in five fractions. The doses were more variable for post-operative RT, ranging from 59.4 Gy to 66.6 Gy for negative margins, and 66 to 70 Gy for positive margins. Approximately three quarters of respondents used 60 Gy in 30 fractions for negative margins, and 66 Gy in 33 fractions for positive margins. For patients who received pre-operative RT and had microscopically positive margins, 39.6% responded that they would give a boost RT to the tumor bed, 49.1% said they would not, and 11.3% felt unsure.

Contouring methods

There was no statistically significant difference in contouring preferences for CTV between superficial and deep STS of the extremities (p = 0.18) (Table 2). For both the superficial and deep STS, the majority preferred to use T2 signal abnormality on MR scan with 1.5 cm circumferential and 3 cm superior/inferior margin around GTV, cropping away from the bone. There was a greater proportion of correspondents who use a larger (2 or 3 cm) circumferential margin for superficial STS than for deep STS. Many also cropped away from the fascia and/or uninvolved muscle groups. Two respondents commented on using a different extension for deep margins, such as 1.5 cm or not extending for the deep margin in superficial tumors.

**Table 2:** Preferences for CTV margin for contouring of an extremity STS. Respondents were asked to choose all preferences that apply to them. There was no significant difference between contouring of a superficial versus deep STS, although there was a trend towards the use of larger margins for both circumferential and superior/inferior for deep STS. Cropping away from bone was more important for deep STS. Additional comments by respondents indicated that many also crop out of uninvolved muscle groups, fascia, and other compartment boundaries. CTV: Clinical target volume; GTV: Gross tumor volume; STS: Soft tissue sarcoma.

Preferences for CTV contouring	Superficial STS	Deep STS
	Respondents (%)	Respondents (%)
T2 signal abnormality on MR scan	38 (71.7)	39 (73.6)
1 cm margin circumferentially around GTV	6 (11.3)	6 (11.3)
1.5 cm margin circumferentially around GTV	28 (52.8)	35 (66.0)
2 cm margin circumferentially around GTV	12 (22.6)	8 (15.1)
3 cm margin circumferentially around GTV	6 (11.3)	4 (7.6)
3 cm margin superior/inferior around GTV	22 (41.5)	28 (52.8)
4 cm margin superior/inferior around GTV	12 (22.6)	17 (32.1)
Crop CTV away from bone	38 (71.7)	41 (77.4)

Use of a normal soft tissue strip

A total of 69.8% of respondents contour a normal tissue strip of skin and subcutaneous tissue of an extremity, the majority (61.8%) aiming to cover 20-35% of the circumference (average: 31%). In 86.5% of the cases, the normal tissue strip was contoured at the discretion of the treating oncologist. 91.7% agreed to a statement that the planning target volume (PTV) should preferably include no more than 66-75% of limb circumference. There was a significant variability in dose constraints, with no more than 50% of the normal tissue strip receiving 50 Gy being the most common (43.2%) constraint (Table 3). 13.5% reported not using a constraint routinely.

**Table 3:** Commonly used constraints used for the longitudinal normal tissue strip of the extremities as an organ at risk. The most commonly used constraint was V50% less than 20 Gy, and other constraints while 13.5% of those who contour the normal tissue strip did not routinely set constraints. * Indicates the constraints that were not available as choices but were provided by the respondents by choosing “Other (please specify).”

Constraints for the normal tissue strip	Respondents (%)
No more than 50% of the volume should receive 20 Gy	16 (43.2)
Limit 20% of volume to less than 25 Gy, and 50% of volume to less than 20 Gy	8 (21.6)
No constraint used routinely	5 (13.5)
*As low as achievable	3 (8.1)
*Mean dose less than 20 Gy	2 (5.4)
*Maximum dose less than 30 Gy	1 (2.7)
*Maximum dose less than 20 Gy	1 (2.7)
*No more than 75% of the volume should receive 20 Gy	1 (2.7)

Case scenario

“For a healthy 40-year-old man with a large (almost the entire circumference) non-metastatic sarcoma of the upper arm, if considered limb-salvageable by the surgeon,” 44 correspondents (86.3%) would give pre-operative RT, two (3.9%) post-operative RT, and 10 (9.8%) would recommend an amputation. Other comments included consideration of neoadjuvant chemotherapy combined with pre-operative radiotherapy, and consideration of isolated limb perfusion.

## Discussion

STS of the extremities constitutes approximately 40% of all STS. Use of adjuvant radiotherapy has been widely adopted since at least 1950s to improve local control [8]. Since several important studies that showed the similar magnitude of local control benefits between pre- and post-operative RT, there has been a shift favouring pre-operative RT as it has a lesser chance of severe long-term toxicities compared to post-operative RT [9-12]. The practice of RT for STS of the extremities has also been evolving with newer simulation and treatment techniques, and the objective of the current study was to assess different aspects of the current practice. This study shows that most of the respondents work in an academic setting with multiple cases per year. Not surprisingly, the majority had access to multidisciplinary boards and quality assurance rounds for discussion of challenging cases, if not for every STS case. Consistent with the technological improvements seen within radiation oncology, the results show that newer RT techniques such as VMAT, IMRT, and simulation with MR fusion and MR simulation are widely implemented for treatment of STS of the extremities.

Although pre-operative RT is becoming more popular, this study shows that post-operative RT is still frequently given and there are unique challenges for each type of treatment for contouring and treatment planning. This study shows a wide range of responses regarding the total dose of RT, use of hypofractionation, and use of a higher dose or boost in case of a positive margin. To our knowledge, there has been no recent investigation comparing the effectiveness of hypofractionation to conventional radiotherapy for soft tissue sarcoma of the extremities, but a recent report on use of pre-operative hypofractionated RT (30 Gy in 10 fractions) combined with doxorubicin for primary or recurrent extremity STS showed improved quality of life and functional outcome results one year post treatment [13]. Use of hypofractionation for successful management of other types of tumors that have similar radiobiologic characteristics suggests that hypofractionation for a pre-operative setting may become more prevalent in the future [14, 15].

Although a couple of studies concluded that a boost for a positive margin may not confer any local control benefit, the uncertainties around the efficacy of the extra dose are certainly reflected in the variable doses for post-operative radiotherapy shown in this survey [16, 17]. Further research may be necessary before we can reach a consensus for the use of boost and recommendation of a dose with the optimal therapeutic ratio.

Most protocols for STS of the extremities recommend sparing of a normal tissue strip to prevent lymphedema by not treating the entire circumference. It is believed that lymphedema caused by RT is mostly caused by disturbance of the dermal lymphatics, located 1.5-2 mm from the surface. Attempts to preserve a longitudinal fraction of the limb have been practiced since the 1960s when RT for extremity STS was delivered by 60-Cobalt or electron beam with doses ranging from 63-70 Gy in 2 Gy per fraction. Suit et al. mentions in their 1975 publication that this has been part of their protocol since 1964 as it resulted in marked improvement in the function of the limb, and to our knowledge, this is the first report on the use of a normal tissue strip as a structure/organ to spare [18]. There has been no study that reported on the magnitude of this observed benefit to date.

A commonly used constraint for the longitudinal normal soft tissue strip is no more than 50% receiving 20 Gy, as seen in the RTOG consensus report and a landmark paper that compared pre-operative vs. post-operative RT [9, 19]. Other approaches include leaving the normal tissue strip untreated for at least half of the course unless it reduces target margin to less than 2 cm [9], or sparing this strip as much as possible, limiting to less than 20 Gy [20]. We have been aware that the practice of contouring a normal tissue strip has been disregarded at some institutes, and this survey confirms that considerable (30.2%) respondents do not contour a normal tissue strip. A recent study looking into the dosimetric distribution of megavoltage reported sparing of the superficial layer, which is practically non-existent for cobalt [21]. A recent study at our institution has also shown that if the PTV constraints are met with other ways of controlling dose distribution, such as spatially variant normal tissue objectives, then sparing of the normal tissue strip may be automatically achieved [22]. For the last question of our survey, where the case scenario regarding a large sarcoma involving almost the entire circumference was presented, the majority chose to try pre-operative radiotherapy when deemed salvageable by the surgeon, reflecting the optimistic sentiment despite the inability to spare a normal tissue strip. Based on the current evidence and prevalent use of megavoltage with conformal techniques, discontinuing the use of soft tissue contouring as a normal organ with constraint may be acceptable, especially with a quality assurance round reviewing each plan for safety.

Our study has several limitations. Due to the relatively small sample size (n = 58), our results may not accurately represent the practice around the world, particularly in Asia. To maintain this survey anonymous, the information gathered did not contain any personally identifiable information and there was no way to trace any of the respondents. Therefore, an honour system was employed to request that they complete the survey only once. As our target group was reached via professional societies, this study reached a specific group of radiation oncologists; therefore, selection bias needs to be factored in when interpreting the results.

## Conclusions

Major aspects of RT planning for extremity STS were similar among the respondents, most of whom were academically affiliated seeing greater than 20 STS cases per year. Over twice as many employed pre-operative as opposed to post-operative RT. Compared to the dose-fractionation regimens where there appears to be a consensus for both pre-operative and post-operative settings, there was greater heterogeneity in planning details such as the use of margins for CTV contouring, use of normal tissue strip as an avoidance structure, and RT boost for positive margins for patients who received pre-operative radiotherapy. This survey identifies the area of variable patterns of practice and provides insight into the areas for further discussion and/or research before consensus can be reached.

## Appendices

Please follow the link to see the survey questions.

https://www.surveymonkey.com/r/Preview/?sm=8mcXsoEa9WezRheqRYM9sNIQm6M2y0GttI6jXNglnKlNtzRs9ypmK610Q35qvdYz
